# Effect of gaze direction recognition task on pain, rom and functional activities in cervicogenic headache patients

**DOI:** 10.1186/s12883-025-04405-z

**Published:** 2025-10-15

**Authors:** Mohamed Abdelaziz Emam, Salma Ragab, András Attila Horváth, Olfat Ibrahim Ali, Zizi M. Ibrahim, Magda Ramadan

**Affiliations:** 1 Basic Sciences Department, Faculty of Physical Therapy, Kafr Elsheikh University, Kafr Elsheikh, 33511 Egypt; 2 János Szentágothai Neurosciences Division, Semmelweis University, Budapest, 1085 Hungary; 3 Department of Neuropsychiatry, Faculty of Medicine, Kafr Elsheikh University, 33511 Kafr Elsheikh, Egypt; 4 Neurocognitive Research Centre, Nyírő Gyula National Institute of Psychiatry and Addictology, Budapest, 1135 Hungary; 5 Department of Anatomy, Histology and Embryology, Semmelweis University, Budapest, 1094 Hungary; 6 Research Centre for Natural Sciences, Hungarian Research Network, Budapest, 1117 Hungary; 7 Physical Therapy Program, Batterjee Medical College, Jeddah, 21442 Saudi Arabia; 8 Department of Rehabilitation Sciences, College of Health and Rehabilitation Sciences, Princess Nourah bint Abdulrahman University, P.O. Box 84428, Riyadh, 11671 Saudi Arabia; 9 Basic Sciences Department, Faculty of Physical Therapy, Cairo University, Giza, 12613 Egypt

**Keywords:** Proprioception, Cervicogenic headache, Neck pain, Motor imagery, Pain measurement

## Abstract

**Background:**

Cervicogenic headache is characterized by unilateral headache potentially stemming from cervical spine mechanical dysfunction. Research indicates that proprioceptive exercises, specifically gaze direction recognition (GDR), are found to be effective in reducing cervical joint position error and enhancing the quality of cervical afferent signals to the central nervous system.

**Purpose:**

This study aimed to evaluate proprioceptive training’s impact on headache pain intensity, functional limitation, and neck motion range in individuals experiencing cervicogenic headache.

**Subjects and methods:**

This study employed a randomized controlled design involving 40 participants with cervicogenic headache between 35 and 49 years of age, divided equally into two groups. CONT (control) received only conventional physical therapy interventions, while EXPR (Experimental) underwent both proprioceptive training and standard physical therapy. Both programs consisted of 24, 60–70 min long sessions over 8 weeks. Assessment tools included the Numeric Rating Scale for headache pain, the Neck Disability Index for functional limitation evaluation, and a cervical range of motion (CROM) device for mobility assessment.

**Results:**

Statistical analysis showed that headache pain and disability level significantly decreased (*P* = 0.0001) post-intervention in both groups, with superior outcomes in EXPR. Similarly, cervical mobility significantly improved (*p* = 0.0001) in both groups following treatment, with EXPR demonstrating greater enhancements.

**Conclusions:**

Gaze direction recognition exercise (GDR) is effective in reducing headache pain severity, and disability level, and increasing cervical ROM in subjects with Cervicogenic headache.

**Trial registration:**

Approval was granted on 29 February 2024. (PACTR202402489039282), available at https://pactr.samrc.ac.za/.

**Supplementary Information:**

The online version contains supplementary material available at 10.1186/s12883-025-04405-z.

## Introduction

Cervicogenic headache (CGH) typically manifests as one-sided pain potentially originating from structural impairment of the cervical spine, particularly involving the top three cervical segments. This condition may be associated with limited neck mobility and increased sensitivity in the occipital-cervical region, often resulting from cervical vertebral deterioration and damage to surrounding soft tissues [1].

Headache disorders affect approximately 47% of people worldwide, with CGH accounting for 15–20% of persistent and recurring headache cases. CGH has a prevalence of 2.2–2.5% among adults [2]. In clinic-based studies, the relative frequency among headache subjects is about 3.1%, with women representing 80.8% of cases [3]. The average age of CGH onset is around 43 years, and the condition is about four times more prevalent in women than men [4].

The superior part of the cervical spine contains a higher concentration of proprioceptive receptors compared to its lower regions [5]. Effective coordination of sensory information from visual, vestibular, somatosensory, and cervical receptor systems plays a crucial role in maintaining postural equilibrium [6].

Accurately determining head position relative to both spatial coordinates and the trunk requires integration of multiple sensory inputs beyond vestibular and visual signals specifically proprioceptive feedback from the cervical region. This proprioceptive information originates from diverse structures surrounding the cervical spine, including muscular tissue, articular components, and cutaneous receptors. Conscious proprioceptive awareness is fundamental for optimal joint performance during athletic activities, everyday functions, and occupational tasks. When proprioceptive feedback mechanisms are compromised, whether through experimental manipulation or pathological, conditions notable deficiencies in motor control typically emerge [7].

Non-pharmacological treatments, especially manual ther- apy and targeted physiotherapy, are increasingly recognized as important options in the management of different types of headaches. Many patients with primary headaches present with musculoskeletal problems, such as altered posture of the head and spine or restrictions in cervical joint mobility. These dysfunctions are not just a cause of local pain, but they also may be a reason of peripheral sensitization. This central sensitization process is the reason that the headache symptoms are here to be amplified over time. A recent study by Deodato et al. has shown that people with chronic migraine and tension-type headache often share similar pat- terns of postural and spinal dysfunction compared to healthy individuals, underlining the role of musculoskeletal factors in headache disorders [8].

Similarly, a review by Liang et al. (2019) found that tension-type headache is commonly associated with forward head posture and loss of cervical movement, while people with migraine indicate only slight but still statistically significant neck function impairments [9]. These cervical impairments may stimulate nociceptive pathways, contributing to peripheral sensitization in the short term and central sensitization in the long term, which in turn helps maintain chronic headache. This knowledge makes the case for interventions that do not just temporarily relieve pain more compelling. Hence, these interventions are also expected to cover sensorimotor dysfunctions as the core that cause the continuation of headache.

Proprioceptive training exercises are found to be effective in reducing cervical joint position error and enhancing the quality of sensory information transmitted from cervical structures to the central nervous system through specialized exercises incorporating repetitive, targeted contractions of the cranio-cervical muscles, areas with concentrated muscle spindles, whether through precision based repositioning practice or exercises focusing on coordinated eye-head movements [10].

Based on current literature, evidence on the effects of proprioceptive training in CGH is still limited. Emam et al., (2024) previously demonstrated that gaze direction recognition (GDR) training improved postural stability and reduced pain in patients with CGH [11]. However, that study did not investigate other clinically relevant outcomes such as cervical range of motion and neck-related disability in CGH subjects.

Building on these findings, the present study aimed to evaluate the effectiveness of proprioceptive exercises—specifically GDR protocol where participants observed another person’s neck rotation from a posterior view and attempted to identify gaze direction. This methodology required participants with CGH to engage in guided mental movement visualization techniques. It incorporates motor imagery techniques, with prior research demonstrating that GDR exercises substantially elevate oxyhemoglobin levels in both the premotor cortex and superior temporal sulcus regions compared to passive observation of movement [12].

Control of gaze direction involves the coordinated action of both eye and neck movements. In subjects with chronic neck pain, sensory-motor conflict may arise due to incongruence between expected and actual sensory input from the cervical region, particularly when combined with visual-motor discrepancies during gaze-related tasks. This incongruence can contribute to altered proprioceptive function and persistent symptoms [12].

Our intervention incorporates proprioceptive exercises and graded motor imagery (GMI), both of which have been shown to improve joint position sense and proprioceptive acuity. GMI activates higher-order motor areas, while mirror therapy—part of the GMI approach—engages the primary sensory-motor cortex, helping to restore coherent sensory and motor integration by enhancing visual-motor feedback and facilitating motor planning [13]. Furthermore, clinical studies support the effectiveness of such interventions in subjects with chronic neck pain. For example, Nobusako et al. (2012) demonstrated that a gaze direction recognition task can improve both pain and cervical range of motion [12].

Although several studies have explored the benefits of manual therapy and general physiotherapy in patients with CGH, evidence regarding interventions specifically targeting cervical proprioception remains limited. Proprioceptive training in the form of GDR has shown promise in improving joint position sense and reducing pain in chronic neck pain populations [12], but research on the use of this protocol in patients with CGH is limited, particularly regarding its effects on cervical range of motion, headache pain intensity, and functional impairment, which are key clinical outcomes in the management of CGH.

Therefore, this study aimed to address this gap by:

- Primary aim: To evaluate whether adding GDR to standard physiotherapy is more effective than physiotherapy alone in reducing headache pain intensity, neck-related disability, and improving cervical range of motion in patients with cervicogenic headache.
- Secondary aim: To determine whether GDR improves proprioceptive performance (gaze recognition accuracy and reaction time) compared to baseline measurements.

## Materials and methods

### Design

This research utilized a forward-looking, randomized, controlled experimental design. The investigation took place over a 16-month period from November 2023 through February 2025. Prior to participation, each subject received a comprehensive explanation of the research methodology and provided written informed consent. This study was conducted in accordance with the Declaration of Helsinki code of ethics and was approved by the Ethics Committee of Cairo University, Faculty of Physical Therapy (approval no.: P.T.REC/012/004977). Additionally, the investigation was subsequently registered with the Pan African Registration Trials system (PACTR202402489039282) *||* (https://pactr.samrc.ac.za/). The approval was granted on 29 February, 2024.

## Participants

**Fig. 1:**
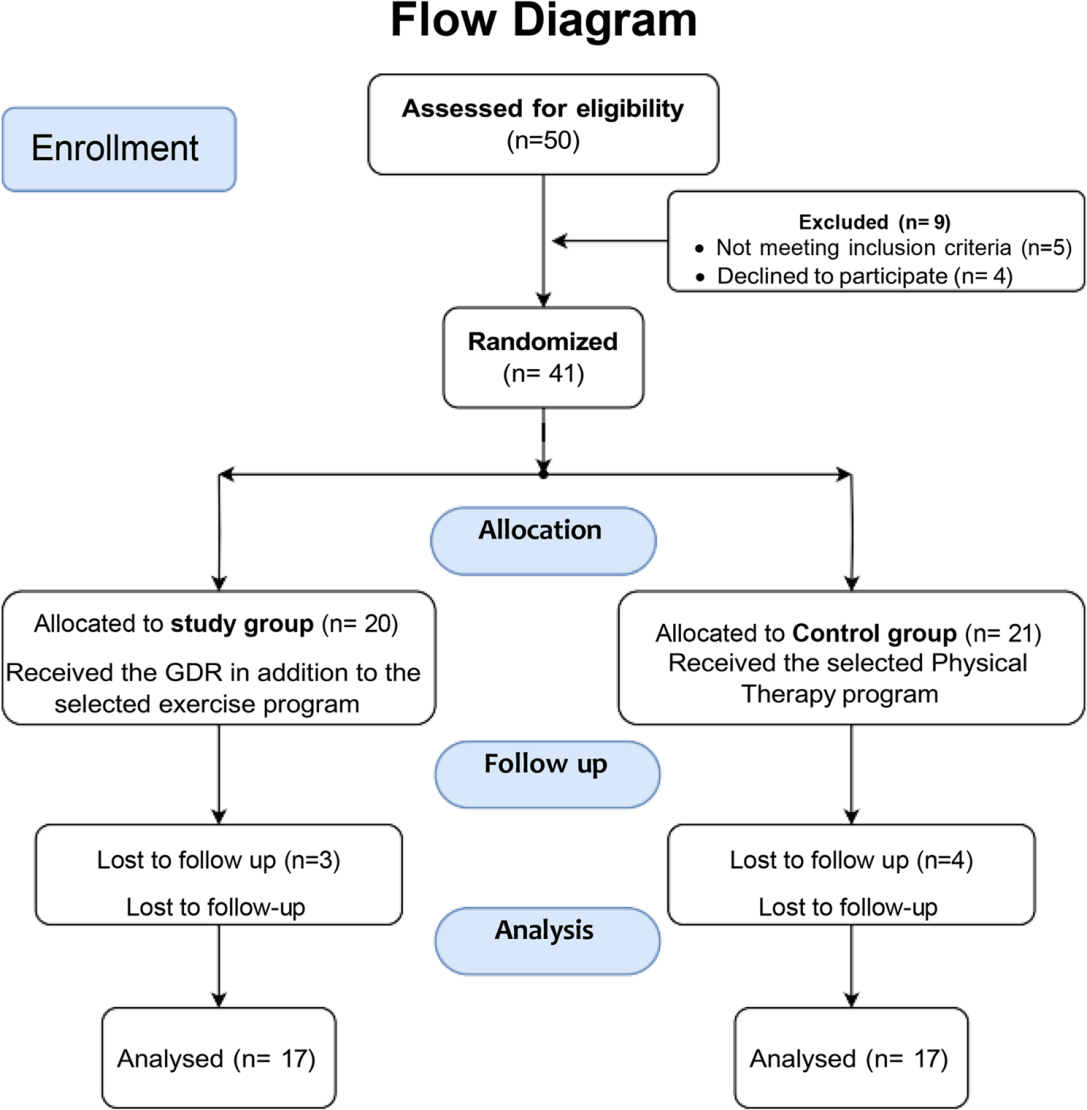
Flowchart of patient recruitment and study participation

The investigation enrolled 50 individuals from two medical facilities in Egypt: Kafr Elsheikh University Hospital and Kafr Elsheikh General Hospital. Participants qualified for inclusion if they met established diagnostic criteria for cervicogenic headache (CGH) [14]. Eligible participants were between 35 and 49 years old and were diagnosed with CGH according to the diagnostic criteria of the International Classification of Headache Disorders, 3rd edition (ICHD- 3) [14, 15] headache pain originating in the neck that radiated to the frontotemporal area, pain that worsened with neck movement, limited cervical range of motion, tenderness in at least one upper cervical spine joint (C1-C3), and recurring headaches occurring at minimum once monthly for the previous year. The research team excluded potential participants who reported any prior head or neck surgery or injury, existing musculoskeletal conditions, neurological disorders, metabolic syndromes, abnormal blood pressure (either high or low), vestibular disorders, or inner ear inflammation. Figure 1.

### Randomization

Participant allocation was conducted through basic random- ization methods using sealed opaque envelopes. An unaffil- iated individual managed the allocation process by having participants select envelopes from a container in sequential numerical order. Each envelope contained a designation letter that assigned the participant to either the experimental group (EXPR) or the control group (CONT). subjects were not informed which one was the experimental group, this ran- domization procedure ensured unbiased distribution between the two comparable treatment groups. Outcome assessments were performed by an investigator who was blinded to group assignment and was not involved in delivering the treatments. Because of the nature of the interventions, the treating therapist could not be blinded; however, they were not involved in the evaluation or data analysis.

### Interventions

#### Control group

Participants received selected physical therapy rehabilitation (SPT). Each session of the selected physical therapy (SPT) program began with the application of a hot pack to the neck and shoulder region for 20 min, followed by 20 min of transcutaneous electrical nerve stimulation (TENS) using conventional settings (typically 50 Hz frequency and 100 µs pulse duration) applied to the cervical area. Ultrasound therapy was then administered for 5 min to the affected neck muscles, generally at an intensity of 1–1.5 W/cm² in continuous mode [16, 17]. In addition to these modalities, participants performed therapeutic exercises targeting cervical range of motion, postural correction, and isometric strengthening of the neck muscles. These exercises were prescribed as 10 repetitions per set, to be completed three times daily. This comprehensive program was designed to address pain, improve cervical mobility, and enhance overall function in individuals with CGH [18, 19]. (see Appendix (1))

#### Experimental group

These participants received the selected physical therapy rehabilitation protocol administered to Control group, supplemented with an additional 10-minute session of gaze direction recognition (GDR)exercise during each treatment visit. This innovative GDR technique represents a novel therapeutic approach designed to enhance cervical muscle proprioceptive awareness and facilitate rehabilitation for individuals experiencing neck dysfunction.

#### GDR exercise protocol

The experimental tool featured a rectangular Table (1800 mm × 400 mm) positioned 75 cm from the investigator, with six sequentially numbered blocks arranged at equal intervals across its surface. Participants maintained visual access to all blocks throughout the procedure. The investigator redirected their gaze toward randomly selected numbered blocks through coordinated eye movements and neck rotation, initiating each directional change following a predetermined signal from a research assistant. The investigator maintained fixed visual focus on each selected block until receiving the participant’s response. During this process, participants observed the investigator’s neck rotation from a posterior position and were instructed to mentally visualize the target of the investigator’s gaze. Participants were instructed to: “Observe the therapist’s neck and head movement carefully. Imagine yourself performing the same head movement while keeping your body still, only rotate your neck to follow the direction of the therapist’s movement. Focus on how your neck muscles would move to follow the same gaze direction, and verbally indicate the block number you believe the therapist is looking at.” Participants then verbally identified which block they believed the investigator was observing, responding as promptly as possible [12].

The task difficulty was gradually increased over the eight- week period by: [1] reducing the time between trials, and [2] decreasing verbal cues. Participants were seated in a supported chair with feet flat on the floor. They were instructed to keep their trunk stationary during head movements. An assistant therapist observed each session and provided immediate correction if any compensatory trunk or body movements occurred. Trials were repeated if the posture was not maintained.

**Fig. 2:**
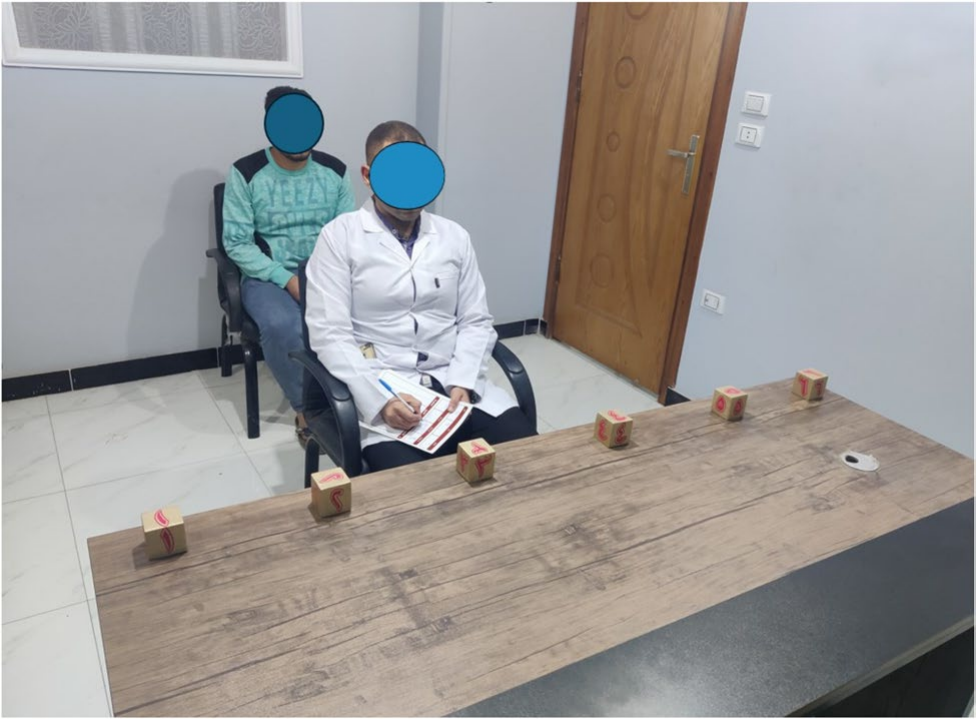
Experimental design of gaze direction recognition task. Each column represents the positional relationship between a subject and an experimenter with six numbered boxes

Participants received no immediate accuracy feedback until the conclusion of the entire experimental session. A research assistant documented both response accuracy and reaction times throughout the procedure. Each complete GDR assessment consisted of 30 consecutive trials following this protocol, typically requiring approximately 10 min to complete. Participants were explicitly instructed to remain stationary during the assessment, permitting only neck rotation to track the investigator’s directional movements [12] as Fig. 2.

### Outcome measures

A. Primary outcomes.

1. Pain severity: The Numeric Rating Scale (NRS) is an alternative to the Visual Analogue Scale (VAS) commonly used for measuring respondents’ perceived pain levels. The NRS provides specific predetermined categories for respondents to rate their headache pain. The NRS usually uses an 11-point rating system, with 0 denoting ”no headache” and 10 denoting the “worst headache” [20].This method simplifies the rating process and is often more accessible for participants, while still maintaining strong validity and reliability in headache pain assessment [21, 22].
2. Neck disability Function was evaluated using the Neck Disability Index (NDI), a comprehensive assessment tool containing ten categories: pain intensity, self-care ability, lifting capacity, reading tolerance, headache frequency, concentration ability, work performance, driving capability, sleep quality, and recreational participation. Participants selected from six graduated response options (scored 0–5) for each category that most accurately reflected their current condition [23]. The examiner calculated the total score by summing all responses and dividing by the maximum possible points (50, or 45 if one category remained unanswered), yielding values ranging from 0 (indicating absence of pain or functional limitation) to 50 (signifying severe pain and functional impairment) [24, 25]. This raw score was subsequently converted to a percentage by multiplying by 100 (final formula: [score/50] × 100 = percentage of disability). A score of 10–28% is mild disability, 30–48% is moderate, 50–68% is severe and 72% or more is complete [26].
3. Cervical Range of Motion (ROM): Active cervical ROM was measured using a Cervical Range of Motion (CROM) device (Performance Attainment Associates, St. Paul, MN, USA), a validated and reliable tool for assessing cervical mobility in clinical and research settings [27, 28]. The device uses inclinometers and a compass to accurately record active flexion, extension, lateral flexion, and bilateral rotational movements.

Prior to measurement, participants were instructed to remove potential obstructions including eyewear, headwear, and accessories. They donned appropriate attire (T-shirt or sleeveless top) and secured any hair that might interfere with observation of the neck region. Measurements were taken with participants seated in a straight-backed chair with feet rested flat on the floor and upper extremities positioned at the sides with shoulder relaxed. The CROM was strapped to participant head and then they were asked to place their head in neutral position to be 0. Two magnet bars were placed on the neck anteriorly and posteriorly using a soft belt to adjust the CROM compass. Additionally, they viewed a brief demonstration of all six cervical movement patterns to be assessed. To enhance measurement reliability and minimize positional variables, standardized protocols were implemented to establish consistent body alignment and a defined neutral head-neck position (anatomical orientation) before initiating each directional movement assessment.

B. Secondary outcomes

For participants in the GDR exercise group, two outcome measures were assessed: response speed and recognition accuracy. Recognition precision was calculated as a percentage (correct responses divided by total responses, multiplied by 100). Response latency was measured as the time interval between the assistant’s initiation signal and the participant’s verbal identification, using a stopwatch with millisecond precision. For analytical purposes, only reaction times from accurately identified gaze directions were included in the subsequent data analysis.

### Statistical analysis

We evaluated differences in age between groups using an unpaired t-test, while sex distribution was compared with the chi-squared test. To confirm normal data distribution, we employed the Shapiro-Wilk test, and Levene’s test was utilized to assess variance homogeneity across groups. Since our data exhibited normal distribution patterns, we proceeded with parametric statistical analyses. To examine the treatment’s impact on Numeric Rating Scale (NRS) scores, Neck Disability Index (NDI), and cervical range of motion (ROM), we implemented a mixed multivariate analysis of variance (MANOVA).

A priori power analysis was conducted using G*Power. The sample size calculation was computed based on the pain intensity outcome measured using the numerical pain rating scale (NPRS). The assumed effect size was 0.25, corresponding to a medium effect size with 80% power and an alpha level of 0.05. The minimum number in each group was 17. To account for the dropout of 20%, the enrollment started with 20 participants per group. The standard deviation of NRPS ranged from 0.86 to 1.42, indicating moderate variability. For multiple comparisons following the main analysis, we applied post-hoc tests with Bonferroni adjustment. Statistical significance was established at p *<* 0.05. We conducted all statistical procedures using IBM SPSS Statistics (version 25 for Windows, IBM SPSS, Chicago, IL, USA).

## Results

### Subject characteristics

Table 1 showed the subject characteristics of EXPR group and CONT. There was no significant difference between groups in age and sex distribution (p *>* 0.05) (Table 1).

**Table 1:** Demographic data values are shown as mean *±* SD

	CONT	EXPR		t/χ^2^ value	*p*-value
	Mean ± SD	Mean ± SD	MD		
Age (years)	41.20 ± 4.80	39.85 ± 4.50	1.35	t = 1.25	0.22
Sex, n (%)					
Females	10 (58.8%)	9 (52.9%)		*χ*^2^ = 1.45	0.23
Males	7 (41.2%)	8 (47.1%)			
Height (cm)	167.5 ± 6.2	168.8 ± 5.9	−1.3	t = −0.61	0.55
Weight (kg)	72.4 ± 8.5	74.1 ± 7.9	−1.7	t = −0.76	0.45
BMI (kg/m²)	25.8 ± 2.4	26.1 ± 2.3	−0.3	t = −0.39	0.70

*SD* Standard deviation; *MD* mean difference; *BMI* Body Mass Index; *χ*^2^, Chi-squared value; *p*-*value* Probability value

### Effect of treatment on numeric rating scale (NRS), neck disability index (NDI) and cervical range of motion (ROM)

The mixed multivariate analysis of variance revealed a significant interaction between treatment and time factors (F = 7.52, *p* = 0.001, partial eta squared = 0.71). Analysis further demonstrated a significant main effect attributable to treatment intervention (F = 4.67, *p* = 0.001, partial eta squared = 0.97) as well as a significant main effect related to time (F = 138.64, *p* = 0.001, partial eta squared = 0.95).

**Fig. 3:**
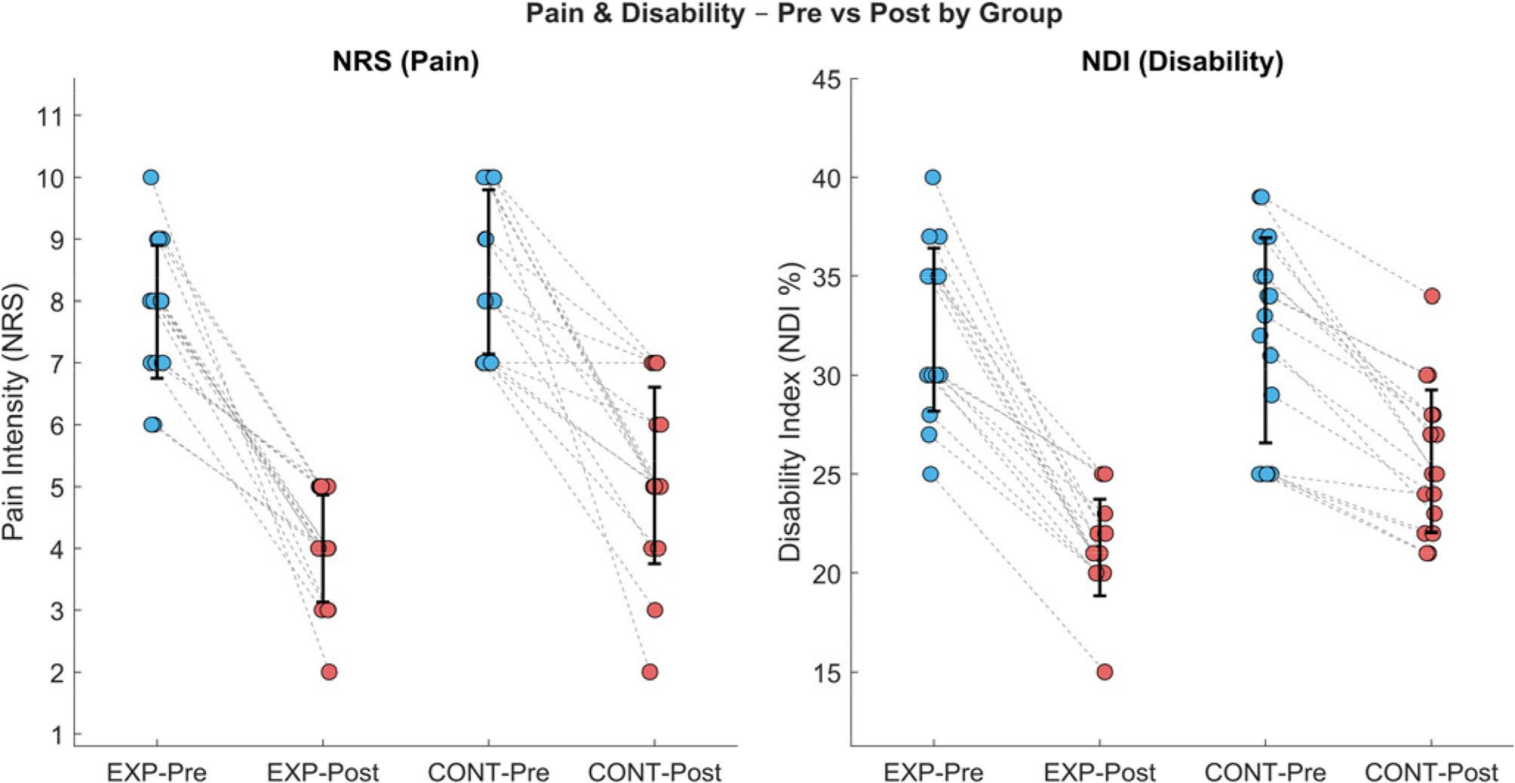
Changes in pain intensity and disability before and after intervention for experimental (EXP) and control (CONT) groups

Both groups exhibited significant reductions in pain intensity measured by NRS, with the control group (CONT) decreasing from 8.47 ± 1.32 to 5.18 ± 1.42 (38.8% improvement, *p* < 0.001), while the experimental group (EXPR) showed a larger reduction from 7.82 ± 1.07 to 4.00 ± 0.86 (48.9% improvement, *p* < 0.001). Similarly, the Neck Disability Index (NDI) improved from 31.8 ± 5.2% to 25.7 ± 3.6% (19.2% reduction, *p* < 0.001) in CONT, and from 32.3 ± 4.1% to 21.3 ± 2.4% (34.1% reduction, *p* < 0.001) in EXPR. These results are summarized in (Table 2) and Fig. 3.

**Table 2:** Mean NRS and NDI pre and post treatment of CONT and EXPR

	Pre treatment	Post treatment			
	Mean ±SD	Mean ±SD	MD	% of change	*p* value
NRS					
CONT	8.47 ± 1.32	5.18 ± 1.42	3.29	38.84	0.001
EXPR	7.82 ± 1.07	4.00 ± 0.86	3.82	48.85	0.001
MD	0.65	1.18			
	*p* = 0.12	*p* = 0.007			
NDI (%)					
CONT	31.76 ± 5.17	25.65 ± 3.58	6.11	19.24	0.001
EXPR	32.29 ± 4.12	21.29 ± 2.44	11	34.07	0.001
MD	−0.53	4.36			
	*p* = 0.74	*p* = 0.001			

*SD* Standard deviation; *MD* Mean difference; *p value* Probability value

Post-treatment assessments revealed significant improvements in cervical range of motion for participants in both treatment groups when compared with pre-intervention measurements (*p* *<* 0.001). In CONT, percentage improvements in movement capabilities were documented as follows: flexion (30.38%), extension (28.98%), right lateral bending (21.32%), left lateral bending (29.71%), right rotation (15.25%), and left rotation (18.01%). EXPR demonstrated more substantial functional gains across all movement parameters: flexion (55%), extension (49.55%), right lateral bending (35.41%), left lateral bending (49.94%), right rotation (22.89%), and left rotation (25.54%).

These findings are detailed in Table 3.

**Table 3:** Mean cervical ROM pre and post treatment of CONT and EXP

ROM (degrees)	Pre treatment	Post treatment			
	Mean ± SD	Mean ± SD	MD	% of change	*p* value
Flexion					
CONT	35.06 ± 3.26	45.71 ± 4.04	−10.65	30.38	0.001
EXPR	33.59 ± 3.58	52.06 ± 4.65	−18.47	55.00	0.001
MD	1.47	−6.35			
	*p* = 0.22	*p* = 0.001			
Extension					
CONT	31.47 ± 8.24	40.59 ± 6.34	−9.12	28.98	0.001
EXPR	30.88 ± 7.95	46.18 ± 3.76	−15.3	49.55	0.001
MD	0.59 *p* = 0.83	−5.59 *p* = 0.004			
Right bending					
CONT	35.88 ± 4.04	43.53 ± 2.34	−7.65	21.32	0.001
EXPR	34.06 ± 4.56	46.12 ± 2.23	−12.06	35.41	0.001
MD	1.82 *p* = 0.22	−2.59 *p* = 0.002			
Left bending					
CONT	32.88 ± 3.33	42.65 ± 3.58	−9.77	29.71	0.001
EXPR	31.82 ± 2.85	47.71 ± 2.68	−15.89	49.94	0.001
MD	1.06 *p* = 0.32	−5.06 *p* = 0.001			
Right rotation					
CONT	52.47 ± 5.35	60.47 ± 5.29	−8	15.25	0.001
EXPR	52.94 ± 4.53	65.06 ± 3.38	−12.12	22.89	0.001
MD	−0.47 *p* = 0.78	−4.59 *p* = 0.005			
Left rotation					
CONT	52.59 ± 5.21	62.06 ± 3.09	−9.47	18.01	0.001
EXPR	53.18 ± 3.84	66.76 ± 3.51	−13.58	25.54	0.001
MD	−0.59 *p* = 0.71	−4.7 *p* = 0.001			

*SD* Standard deviation; *MD* Mean difference; *p value* Probability value

### Effect of treatment on gaze direction recognition (GDR) accuracy and time

**Fig. 4:**
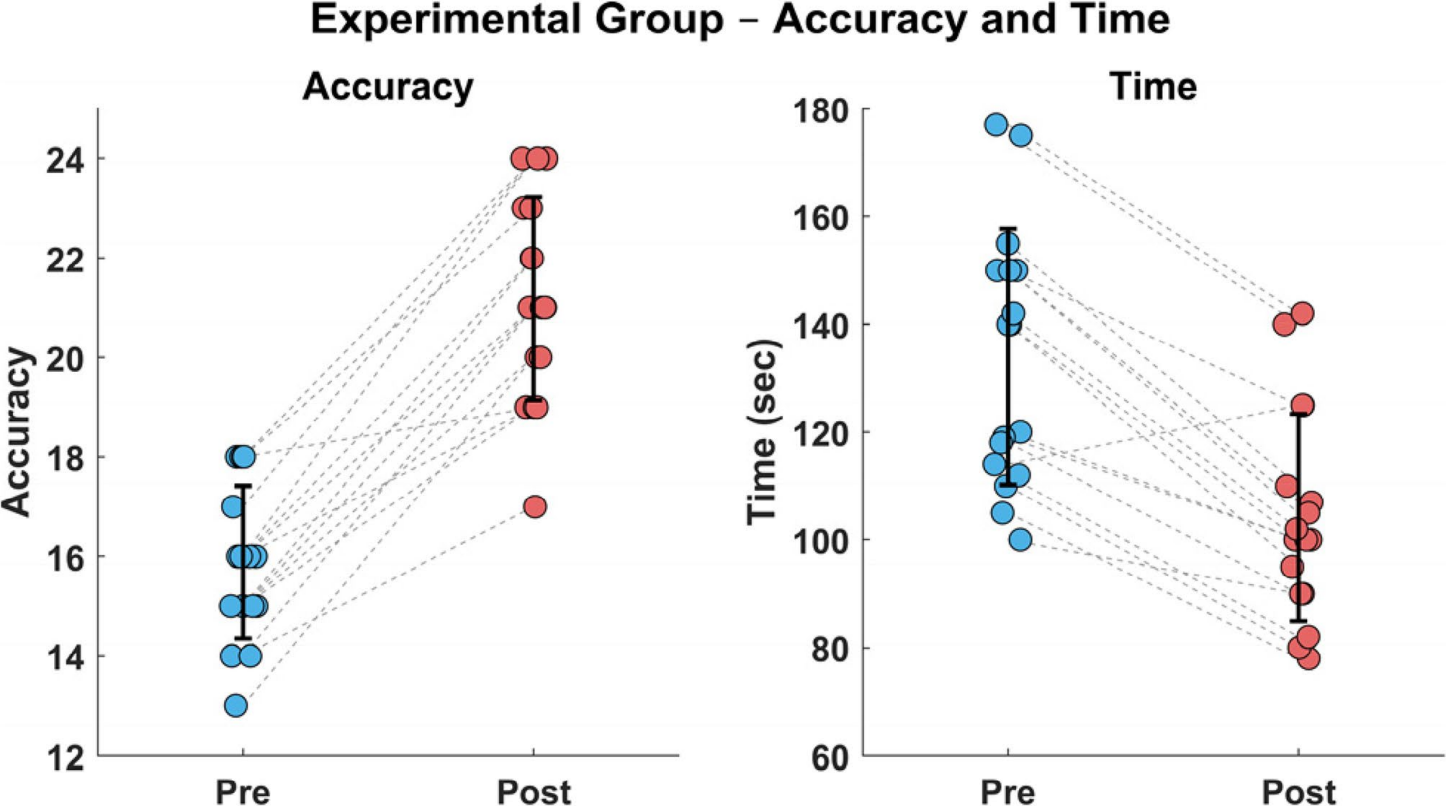
Improvements in GDR Accuracy and time before and after intervention for experimental (EXP) group

Analysis demonstrated significant improvements in both response time and precision following the treatment protocols compared to baseline assessments (p *<* 0.001). In the experimental group, the mean response time decreased from 133.9 ± 23.7 s at baseline to 104.2 ± 19.3 s post- treatment (22.2% improvement, p *<* 0.001), while accuracy improved from 52.9 ± 5.1% to 70.6 ± 6.8% (17.6-point increase, p *<* 0.001). These findings indicate that participants not only performed GDR task more quickly but also achieved markedly higher accuracy after the intervention, these performance metrics are comprehensively presented in Table 4; Fig. 4.

**Table 4:** Mean GDR accuracy and time pre and post treatment of CONT and EXPR

	Pre treatment	Post treatment			
	Mean ±SD	Mean ±SD	MD	t- value	*p* value
Time (sec)	133.94 ± 23.72	104.17 ± 19.25	29.77	8.37	0.001
Accuracy	52.94 ± 5.12	70.58 ± 6.79	17.64	12.16	0.001
*SD* Standard deviation; *MD* Mean difference; *p value* Probability value					

## Discussion

The current study aimed to investigate the proprioceptive training effects in the form of gaze direction recognition (GDR) on pain levels, cervical Range of Motion (ROM), neck function as well as GDR accuracy and time, following the intervention in subjects with cervicogenic headache.

The results demonstrate significant improvements in all four outcome measures following the intervention. These findings hold significant implications for the management and rehabilitation of individuals with this condition.

The mixed multivariate analysis of variance revealed significant interaction between treatment and time factors, indicating a strong influence of the proprioceptive training intervention on pain levels and neck disability. This suggests that the intervention effectively contributed to reducing pain severity and improving functional ability in individuals with cervicogenic headaches. The within-group comparisons further support this, showing significant decreases in NRS and NDI scores post-treatment for both CONT and EXPR.

The change percentage in NRS and NDI scores indicates the magnitude of improvement, with EXPR exhibiting a higher percentage change compared to CONT. This implies that EXPR may have experienced a more pronounced reduction in pain and disability, potentially due to specific characteristics or responsiveness to the intervention [29, 30]. Investigating the effects of cervical region injury, operative intervention, and proprioceptive activities during clinical evaluation offers insights into the intricate neural mechanisms that facilitate motor skill acquisition [31]. With enhanced comprehension of pain mechanisms, preventative physical therapy interventions—particularly those targeting cervical spine health—have received increased emphasis in contemporary clinical practice [32].

Abnormal cervical inputs observed as a result of pain, inflammation, changing muscle fiber severity, and related changes lead to changes in sensorimotor arrangement and timing in subjects with CGH. Therefore, the relationships among pain intensity, neck disability level, eye coordination, balance, and proprioception have been investigated in various studies [33–35].

Active patient participation in therapeutic interventions has become a focus of re-search, in contrast to conventional passive treatment approaches [36]. Among the various methods requiring patient engagement, proprioceptive techniques appear particularly effective as they directly stimulate important proprioceptors including those in joints, muscles, and golgi-tendon organs [37].

Research by Jull and colleagues demonstrated that individuals suffering from chronic neck pain (CNP) who underwent proprioceptive training for a three-week period experienced superior outcomes in joint positioning, pain reduction, and perceived neck disability compared to those receiving standard therapy [10, 18]. These findings are supported by recent evidence suggesting that interventions focusing on sensorimotor control can address both peripheral and central sensitization mechanisms that contribute to headache persistence [8]. However, a literature review indicates insufficient evidence to conclusively determine whether incorporating proprioceptive exercises into traditional physical therapy protocols significantly enhances pain management and functional improvement in chronic neck pain cases [38]. Additionally, the vestibular system manipulation induced by head tilt in the treatment group, which affected postural outcomes, may further support the presence of proprioceptive dysfunction as a contributing factor to CNP [39]. The findings from our investigation clearly demonstrate pain reduction with both treatment methods, though proprioceptive training exhibited greater efficacy in alleviating disability perception and improving pain dynamics specifically in subjects with cervicogenic headache (CGH).

Following intervention, our research revealed notable improvements in cervical ROM across both treatment groups. These enhancements were observed in all movement directions, including forward flexion, backward extension, lateral bending to both right and left sides, and rotational movements in both rightward and leftward directions. This indicates that the proprioceptive training program positively impacted the subjects’ ability to move their neck in various directions. The percentage of change in ROM measures highlights the substantial improvements achieved. Once again, EXPR exhibited higher percentage changes compared to CONT, suggesting that individuals in EXPR may have experienced a more significant enhancement in cervical mobility.

 [40, 41].

The between-group comparisons revealed noteworthy differences. EXPR demonstrated significantly greater improvements in NRS scores, NDI scores, and cervical ROM compared to CONT post-treatment. This indicates that subjects in EXPR, with specific characteristics or clinical profiles, may have derived greater benefit from the proprioceptive training intervention. It is important to further investigate the factors contributing to these disparities, which could include baseline pain levels, initial neck function, or individual responsiveness to the intervention.

Research examining cervical traction effects has docu-mented patient-reported neck pain relief occurring up to 12 h following treatment [42] indicating that traction interventions may deliver quick therapeutic benefits. Nev- ertheless, physical therapy approaches typically yield only temporary enhancements in neck pain reduction and mobility range, and these conventional therapeutic techniques prove inadequate for restoring the frequent cervical movements required in everyday activities, as specified in rehabilitation protocols designed for post-cervical injury recovery.

Within the GDR intervention group, accurate cervical movement programming was enhanced alongside rapid peripheral effects from physical therapies (similar to those applied in the control group). This combination likely con- tributed to the sustained therapeutic benefits observed during follow-up assessments conducted days after treatment com- pletion in the GDR group. Progressive improvements in the GDR group notably included significant enhancements in active neck rotation range, whereas the control group lack- ing GDR task implementation failed to demonstrate similar progressive improvements. Additionally, when comparing outcomes between groups, the GDR participants exhibited markedly superior improvement in lateral neck rotation mobility range. The GDR essentially functions as a form of motor imagery training. Such motor imagery has been shown to enhance muscular contraction, improve balance capabilities in older female populations, increase skill pre- cision, optimize movement timing, and reduce hemiparesis symptoms following stroke [43, 44].

Our investigation identified a significant correlation between decreased reaction times and improved response accuracy in participants receiving GDR intervention, along- side enhancements in active cervical motion range. The sequential alleviation of CGH pain and the negative corre- lation observed between NRS pain scores and active motion range measurements in our current research suggest that pain reduction may be directly linked to improvements in move- ment capability [45]. The marked decrease in completion time alongside improved accuracy following treatment in the CGH group is indicative of enhanced motor performance. This finding aligns with the notion that proprioceptive train- ing not only impacts balance but also contributes to improved motor coordination and precision. The reduction in time may reflect increased efficiency in executing movements, while the improved accuracy suggests greater control over fine motor tasks [46]. Comparable beneficial mechanisms probably underlie our current findings, where the GDR pro- duced progressive CGH symptom reduction. When subjects observe the researcher’s neck rotation movements, they are prompted to visualize gaze direction and generate precise mental imagery of neck movement. Observing neck rotations performed by a healthy researcher without cervical pathol- ogy may stimulate neural motor imagery and activate cortical representations specific to neck movement [12, 47, 48].

### Clinical implications

The findings of this investigation highlight the possible advantages of incorporating proprioceptive training into the rehabilitation program for individuals with cervicogenic headaches. By targeting proprioceptive deficits, clinicians may be able to significantly alleviate pain, improve neck function, enhance range of motion, and improve proprioceptive skills, ultimately improving the quality of life for these subjects.

### Limitations and future directions

It is crucial to recognize some of this study’s limitations. The comparatively tiny sample size and specific patient population may limit the generalizability of the findings. One limitation of this study is that we did not directly measure proprioception. While we evaluated improvements in the accuracy and timing of the proprioceptive training techniques, we did not include specific measures of.

proprioceptive ability itself. Future studies should consider incorporating direct assessments of proprioception to provide a more complete understanding of its role in clinical outcomes. Additionally, the use of instrumental methods, such as pressure algometry to quantify pressure pain thresholds and tensiomyography to assess muscle contractile properties, could have provided more objective data on neuromuscular and sensory changes. Incorporating these tools in future studies would strengthen the evidence on the physiological effects of gaze direction recognition training. Another limitation of this study is that outcomes were only measured at the start and immediately after the intervention. Because we did not include any follow-up assessments, we cannot say how long the benefits of the treatment might last. Future research should track patients over several months to see whether the improvements in pain, neck function, and mobility are maintained over time.

## Conclusions

Our randomized clinical investigation examining the impact of gaze direction recognition (GDR) exercises on cervical mobility and discomfort in cervicogenic headache (CGH) subjects demonstrated that sequential implementation of these tasks progressively enhances active neck rotation range while diminishing pain symptoms. The findings indicate that gaze direction recognition exercises represent a promising therapeutic intervention for managing CGH conditions.

## Supplementary Information

Below is the link to the electronic supplementary material.

Supplementary Material 1

## Data Availability

Research data from this study can be accessed by submitting a request to the corresponding author.
